# Galectin 3 expression in primary oral squamous cell carcinomas

**DOI:** 10.1186/s12885-017-3920-2

**Published:** 2017-12-29

**Authors:** Manuel Weber, Maike Büttner-Herold, Luitpold Distel, Jutta Ries, Patrick Moebius, Raimund Preidl, Carol I. Geppert, Friedrich W. Neukam, Falk Wehrhan

**Affiliations:** 1 Department of Oral and Maxillofacial Surgery, Friedrich-Alexander University Erlangen-Nürnberg, Glueckstrasse 11, 91054 Erlangen, Germany; 2 Institute of Pathology, Department of Nephropathology, Friedrich-Alexander University Erlangen-Nürnberg, Erlangen, Germany; 3 Department of Radiation Oncology, Friedrich-Alexander University Erlangen-Nürnberg, Erlangen, Germany; 4 Institute of Pathology, Friedrich-Alexander University Erlangen-Nürnberg, Erlangen, Germany

**Keywords:** Oral squamous cell carcinoma, Oral cancer, Galectin 3, Gal3, Macrophage polarization, oscc, M1, M2

## Abstract

**Background:**

Immunologic factors can promote the progression of oral squamous cell carcinomas (oscc). The phylogenetic highly conserved protein Galectin 3 (Gal3) contributes to cell differentiation and immune homeostasis. There is evidence that Gal3 is involved in the progression of oscc and influences the regulation of macrophage polarization. Macrophage polarization (M1 vs. M2) in solid malignancies like oscc contributes to tumor immune-escape. However, the relationship between macrophage polarization and Gal3 expression in oscc is not yet understood. The current study analyzes the association between histomorphologic parameters (T-, N-, L- Pn-status, grading) and Gal3 expression resp. the ratio between Gal3 expressing cells and CD68 positive macrophages in oscc specimens.

**Methods:**

Preoperative diagnostic biopsies (*n* = 26) and tumor resection specimens (*n* = 34) of T1/T2 oscc patients were immunohistochemically analyzed for Gal3 and CD68 expression. The number of Gal3 expressing cells and the ratio between CD68 and Gal3 expressing cells was quantitatively assessed.

**Results:**

In biopsy and tumor resection specimens, the number of Gal3 positive cells as well as the Gal3/CD68 ratio were significantly (*p* < 0.05) higher in T2 oscc compared to T1 cases. In biopsy specimens, a significantly (*p* < 0.05) increased Gal3 expression and Gal3/CD68 ratio was associated with the progression marker lymph vessel infiltration (L1). Tumor resection specimens of cases with lymph node metastases (N+) had a significantly (*p* < 0.05) increased Gal3 expression. Additionally, a high Gal3/CD68 ratio correlated significantly (*p* < 0.05) with higher grading (G3) in tumor resection specimens.

**Conclusion:**

High Gal3 expression in oscc is associated with tumor size (T-status) and parameters of malignancy (N-, L-status, grading). Gal3 might contribute to M2 macrophage mediated local immune tolerance. Gal3 expression shows association with prognosis in oscc and represent a potential therapeutic target.

## Background

TNM classification and grading only partially predict the prognosis of patients with oral squamous cell carcinomas (oscc) and cannot explain individual clinical courses of the disease. For the progression of oscc, genetic mutations which lead to dysregulation of embryonic signaling pathways [1, 2] as well as immunologic factors play a significant role [3, 4]. The phylogenetic highly conserved protein Galectin 3 (Gal3) is an important mediator between cell differentiation and tumor immunity [5, 6] and contributes to the regulation of macrophage polarization [7, 8].

Tumors and their precursor lesions often reveal alterations of Gal3 expression. Accordingly, a possible contribution of Gal3 to the pathogenesis of pancreatic, colorectal, liver and esophageal cancer could already be shown [9]. There is evidence that Gal3 overexpression promotes malign transformation and metastatic spread [10]. Cancer patients showed increased serum Gal3 and a correlation of Gal3 levels with prognosis was detectable [10].

Evidence exists that Gal3 is also relevant for the progression of oscc. Some studies analyzed the role of Gal3 as prognostic factor especially in tongue cancer [11–13]. One immunohistochemical study in patients with tongue cancer showed an association of Gal3 expression with dedifferentiation, metastases and poor prognosis [12]. Another report examined the expression of Gal3 in a cohort of exceptionally young patients with oscc in several locations without identifying a correlation with tumor stage or grading [14]. In addition to invasive squamous cell carcinomas, an increased expression of Gal3 could already be shown in precursor lesions of oral cancer [6, 15].

Due to its contribution to the regulation of macrophage polarization (M1 vs. M2), Gal3 is an important immune modulator [7, 8]. Macrophages are highly plastic cells involved in the formation of the tumor microenvironment and relevant for oscc prognosis [16] and therapy response [17, 18]. In this context M2 polarized macrophages contribute to immune tolerance and promote tumor progression and metastatic spread [18, 19]. Gal3 can shift macrophage polarization towards M2 [7].

Most studies analyzing Gal3 expression in oscc patients were performed in tongue cancer specimens and therefore only represent a subgroup of oscc. Gal3 expression in oscc was not yet analyzed in the context of a macrophage mediated immune-tolerant microenvironment in oscc. As an association of macrophage polarization with histomorphologic [3] and prognostic parameters [16] in oscc has already been shown, a comparative analysis of Gal3 and macrophage infiltration in oscc might elucidate an involved mediator.

Galectins like Gal3 are members of the lectins and are characterized by the ability to bind β-galactosides [20]. Galectins have a phylogenetically conserved structure and are involved in cellular proliferation, survival, adhesion and migration [20, 21]. With two functionally relevant protein domains, Gal3 is an unique member of the galectin family [20]. It can interact with a variety of intracellular and extracellular proteins and is involved in the pathogenesis of fibrotic and malign diseases as well as in immune regulation [20]. Gal3 can be detected in several human cells like immune cells and epithelial cells [10].

Because of its high degree of phylogenetic conservation, its properties in carcinogenesis and regulation of macrophage polarization, Gal3 could be a prognosticator in oscc patients [22, 23]. Moreover, as Gal3 inhibitors like citrus pectin are available, Gal3 could also be a target for molecular immune modulatory cancer therapy [22, 23].

The current study aims to answer the question if Gal3 expression and the ratio of Gal3 positive cells vs. CD68 positive macrophages in diagnostic biopsies and tumor resection specimens of oscc is associated with histomorphologic parameters (T-, N-, L-, Pn-status, grading) of tumor progression.

## Methods

### Patients and tissue harvesting

A consecutively treated oscc patient collective was selected for this retrospective study. The patient selection was described in previous reports [3, 24]. Biopsies and tumor resection specimens from a total of 34 patients histologically diagnosed with primary oscc were analyzed in this study. Tumor resection specimens from 34 patients and biopsies from 26 patients were available. The biopsies from nine cases were not available for analysis or too small for analysis. Because we counted macrophage infiltration in at least 1.1 mm^2^ of carcinoma tissue, specimens with smaller carcinoma fractions were excluded. All patients were treated in 2011 at the Department of Oral and Maxillofacial Surgery of the University Hospital Erlangen. The study protocol was approved by the ethical committee of the University of Erlangen-Nuremberg (Ref.-No. 45_12 Bc). The specimens used in this study were obtained from tissue samples collected for routine histopathologic diagnosis. Each included specimen was judged to be a representative squamous cell carcinoma. In addition to the diagnosis of oscc, the following inclusion criteria were defined: pT1 or pT2 tumors, no restrictions in the grading of the tumor, no adjuvant preoperative radio- or chemotherapy and no distant metastasis at the time of diagnosis. Patients with former radio- or chemotherapy and pT3 and pT4 tumors were excluded. No study-related changes in the patients’ treatments took place.

The patient cohort (*n* = 34) consisted of 11 tongue oscc, 11 patients with a tumor of the floor of the mouth, 8 with alveolar crest carcinomas, 3 with a tumor of the palate and 1 of the cheek. The average age of the patients (23 males and 11 females) was 63 years. The pathohistological classified N-status was N0 in 19 cases and N+ in 15 cases (including all positive N-states). The histological grading was G1 in 2 cases, G2 in 26 cases and G3 in 6 cases. None of the patients in our cohort had distant metastases.

### Immunohistochemical staining and quantitative analysis

The immunohistochemical staining procedure was performed as previously described [3, 24]. The following primary antibodies were used: anti-Galectin 3 (sc-20,157, clone H-160, Santa Cruz, Dallas, Texas, USA) and anti-CD68 (11,081,401, clone KP1, Dako, Hamburg, Germany). An appropriate positive control was included in each series.

The tumor and biopsy sections were completely scanned and digitized using the method of “whole slide imaging”. The scanning procedure was performed in cooperation with the Institute of Pathology of the University of Erlangen-Nürnberg using a Panoramic 250 Flash III Scanner (3D Histech, Budapest, Hungary) in 40× magnification. All samples were digitally analyzed (Case viewer, 3D Histech, Budapest, Hungary). Quality controls were performed using a bright-field microscope (Zeiss Axioskop and Axiocam 5, at 100–400 × magnification).

For each sample, three visual fields showing the highest infiltration rate of positive cells were selected (hot spot analysis). The complete area of all three visual fields of one specimen was between 1.1 and 1.5 mm^2^ (Case viewer, 3D Histech, Budapest, Hungary).

Micrographs of the selected areas were imported into the Biomas analysis software (modular systems of applied biology, Erlangen, Germany) for cell counting. Two regions of interest were defined in the visual fields using the Biomas software: the epithelial tumor compartment and the stromal compartment.

A quantitative analysis was performed to determine the numbers of Galectin 3- and CD68-positive cells in the epithelial tumor compartment and the surrounding stroma. A Gal3 background staining, as it was visible in the epithelial tumor compartment of all cases, was not counted. Cells with strong Gal3 expression significantly surpassing the background expression of epithelial oscc tumor cells were counted as positive. Assessment of the cell density per mm^2^ was performed as previously described [16, 24].

### Statistical analysis

To analyze the immunohistochemical staining, the cell count per mm^2^ was determined as the number of positive cells per mm^2^ of the specimen. Multiple measurements were pooled for each sample group prior to analysis. The results are expressed as the median and standard deviation (SD) and range. Box plot diagrams represent the median, the interquartile range, minimum (Min) and maximum (Max).

Two-sided, adjusted *p*-values ≤0.05 were considered to be significant. The analyses were performed using an ANOVA test with SPSS 22 for Mac OS (IBM Inc., New York, USA).

## Results

### General morphologic considerations

**Fig. 1:**
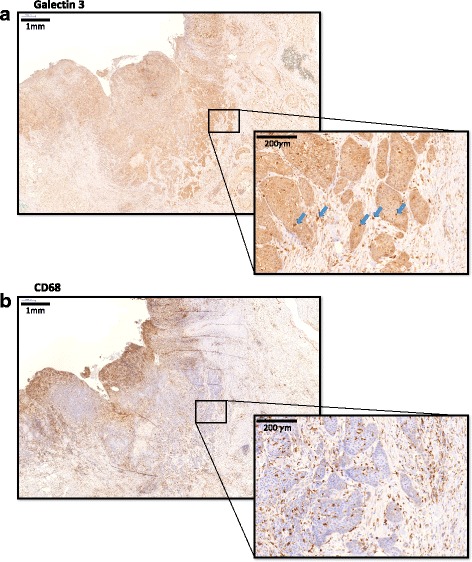
Typical expression pattern of Galectin 3 and CD68 in a tumor resection specimen. The figure shows the typical expression pattern of Galectin 3 positive cells (**a**) and CD68 positive cells (**b**) in an oscc tumor resection specimen. A panoramic view (2.5× magnification) is given on the left side and a magnification of the indicated region (25× magnification) is displayed on the right side. The epithelial carcinoma cells show a weak expression of Gal3. The Gal3 highly positive cells in the epithelial compartment and the stromal compartment were counted for statistical assessment. Five Gal3 highly positive cells are exemplarily marked with an arrow

Galectin 3 (Gal3) was expressed in all investigated specimens. An expression of Gal3 could be observed in all cellular compartments (Fig. 1a). Oscc tumor cells in all patients showed a low level of Gal3 expression. For statistical analysis, the cells with a Gal3 expression significantly surpassing the background Gal3 staining of epithelial ossc tumor cells were counted. These cells could be found in the epithelial tumor compartment as well as in the tumor stroma. The distribution pattern of these Gal3 highly positive cells was similar to the distribution of CD68 positive macrophages (Fig. 1b). The number of CD68 positive macrophages exceeded the number of Gal3 highly positive cells (except in G3 oscc cases).

### Galectin 3 expression in oscc biopsy specimens

**Fig. 2:**
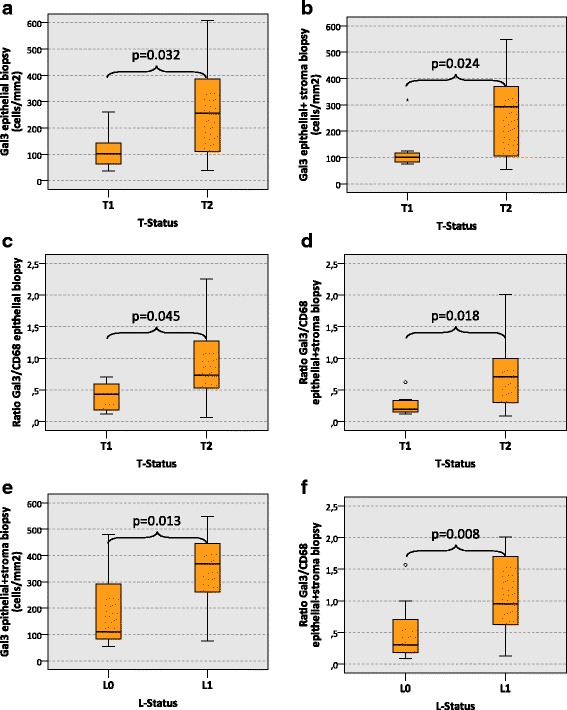
Galectin 3 expression in biopsy specimens of oral squamous cell carcinomas depending on histomorphologic parameters. The **a** and **b** show the expression of Galectin 3 (Gal3) (cells/mm^2^ specimen area) in the epithelial compartment (**a**) and in the whole analyzed specimen area (epithelial + stroma) (**b**) of oscc biopsy specimens depending on the T-status (T1 vs. T2). A significantly increased count of Gal3-positive cells can be found in the epithelial compartment and in the whole analyzed specimen area of T2 oscc biopsy specimens. The **c** and **d** show the ratio between the Gal3 cell count and the CD68 cell count (Gal3/CD68 ratio) in the epithelial compartment (**c**) and in the whole analyzed specimen area (epithelial + stroma) (**d**) of oscc biopsy specimens depending on the T-status (T1 vs. T2). A significantly increased Gal3/CD68 ratio can be found in the epithelial compartment and in the whole analyzed specimen area of T2 oscc biopsy specimens. The **e** and **f** show the expression of Gal3 (cells/mm^2^ specimen area) (**e**) and the Gal3/CD68 ratio (**f**) in the whole analyzed specimen area (epithelial + stroma) of oscc biopsy specimens depending on the L-status (L0 vs. L1). A significantly increased count of Gal3-positive cells and a significantly increased Gal3/CD68 ratio can be found in L1 oscc biopsy specimens. *P*-values generated by the ANOVA test are indicated in all boxplots

Assessing the epithelial compartment of biopsy specimens, the Galectin 3 (Gal3) cell count in T2 oscc cases was significantly higher than in T1 cases (median 256 cells/mm^2^ and 103 cells/mm^2^, respectively, *p* = 0.032) (Table 1, Fig. 2a). Comparably, Gal3 expression in the whole analyzed specimen area (epithelial + stroma) of biopsies was significantly higher in T2 cases compared to T1 cases (median 293 cells/mm^2^ and 101 cells/mm^2^, respectively) (*p* = 0.024) (Table 1, Fig. 2b).

**Table 1:** Galectin 3 (Gal3) cell count (cells/mm2) and Gal3/CD68 expression ratio in oscc biopsy specimens

Gal3 expression in biopsy specimens									
Marker		Gal3 epithelial		Gal3 epithelial + stroma		Ratio Gal3/CD68 epithelial		Ratio Gal3/CD68 epithelial + stroma	
		Median	SD	Median	SD	Median	SD	Median	SD
T-Status	n								
T1	8	103	72	101	80	0.43	0.23	0.19	0.17
T2	17	256	170	293	162	0.73	0.60	0.71	0.61
*p*-value		0.032		0.024		0.045		0.018	
*N*-Status	n								
N0	13	111	174	93	175	0.53	0.61	0.30	0.66
N+	12	290	133	276	125	0.73	0.49	0.68	0.47
p-value		0.143		0.197		0.472		0.511	
L-Status	n								
L0	18	115	132	110	126	0.55	0.41	0.31	0.40
L1	7	323	192	369	169	0.88	0.69	0.95	0.71
*p*-value		0.054		0.013		0.020		0.008	
Pn-Status	n								
Pn0	13	170	138	124	134	0.58	0.43	0.35	0.43
Pn1	9	256	185	344	178	0.73	0.68	0.66	0.71
p-value		0.295		0.184		0.185		0.129	
Grading	n								
G2	16	170	136	179	130	0.59	0.41	0.57	0.44
G3	6	337	188	369	177	1.00	0.75	1.09	0.79
*p*-value		0.120		0.083		0.067		0.088	

Table shows the Galectin 3 (Gal3) cell count (positive cells/mm^2^) and the ratio of Gal3 positive cells and CD68 positive cells in oscc biopsy specimens in view of histomorphologic parameters (T-, N-, L-, Pn-status, grading). Results for the epithelial tumor compartment and the whole analyzed area (epithelial + stroma) are given. Values represent the median, standard deviation (SD) and *p*-value (ANOVA)

The ratio between Gal3-expressing cells and CD68-positive macrophages (Gal3/CD68-ratio) in the epithelial fraction of T2 oscc biopsy samples was significantly higher than in biopsies of T1 cases (median values 0.73 and 0.43, respectively, *p* = 0.045) (Table 1, Fig. 2c). The whole analyzed specimen area (epithelial + stroma) in T2 oscc biopsy specimens revealed a significantly higher Gal3/CD68-ratio than in T1 cases (median value 0.71 and 0.19, respectively, *p* = 0.018) (Table 1, Fig. 2d).

Oscc biopsy specimens in cases with lymph vessel infiltration (L1) showed a significantly higher Gal3 expression compared to L0 cases assessing the whole analyzed specimen area (epithelial + stroma) (median 369 cells/mm^2^ and 110 cells/mm^2^, respectively, *p* = 0.013) (Table 1, Fig. 2e).

The Gal3/CD68-ratio in the whole analyzed specimen area (epithelial + stroma) of biopsies from L1 oscc cases was significantly higher than in L0 cases (median value 0.95 and 0.31, respectively, *p* = 0.008) (Table 1, Fig. 2f). In the epithelial compartment, the Gal3/CD68-ratio in L1 biopsy specimens was also significantly higher than in L0 cases (median value 0.88 and 0.55, respectively, *p* = 0.020) (Table 1).

There was no significant association apparent between Gal3 expression and N-status, Pn-status and tumor grading in oscc biopsy specimens.

### Galectin 3 expression in oscc tumor resection specimens

**Fig. 3:**
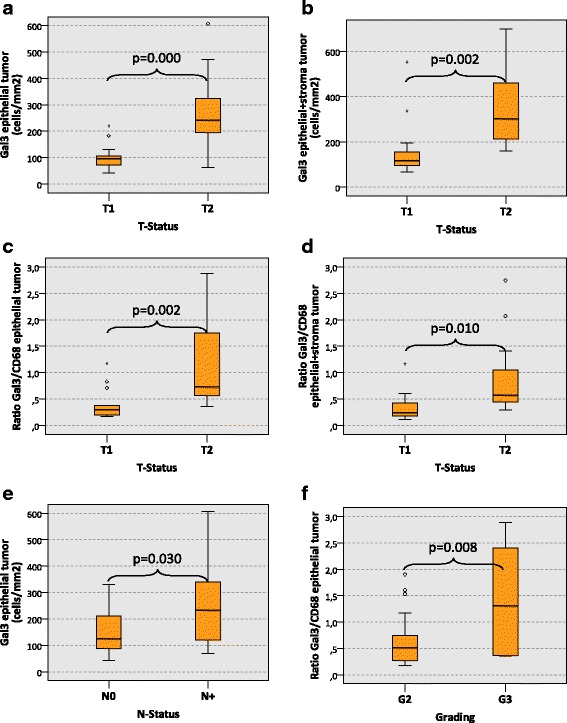
Galectin 3 expression in tumor resection specimens of oral squamous cell carcinomas depending on histomorphologic parameters. The **a** and **b** show the expression of Galectin 3 (Gal3) (cells/mm^2^ specimen area) in the epithelial compartment (**a**) and in the whole analyzed specimen area (epithelial + stroma) (**b**) of oscc tumor resection specimens depending on the T-status (T1 vs. T2). A significantly increased count of Gal3-positive cells can be found in the epithelial compartment and in the whole analyzed specimen area of T2 oscc tumor resection specimens. The **c** and **d** show the ratio between the Gal3 cell count and the CD68 cell count (Gal3/CD68 ratio) in the epithelial compartment (**c**) and in the whole analyzed specimen area (epithelial + stroma) (**d**) of oscc tumor resection specimens depending on the T-status (T1 vs. T2). A significantly increased Gal3/CD68 ratio can be found in the epithelial compartment and in the whole analyzed specimen area of T2 oscc tumor resection specimens. **e** shows the expression of Gal3 (cells/mm^2^ specimen area) in the epithelial compartment of oscc tumor resection specimens depending on the N-status (N0 vs. N+). A significantly increased count of Gal3-positive cells can be found in N+ oscc tumor resection specimens. **f** shows the Gal3/CD68 ratio in the epithelial compartment of oscc tumor resection specimens depending on the grading (G2 vs. G3). A significantly increased Gal3/CD68 ratio is detected in the epithelial compartment of G3 oscc tumor resection specimens. P-values generated by the ANOVA test are indicated in all boxplots

Analyzing the epithelial fraction of tumor resection specimens, the Galectin 3 (Gal3) cell count in T2 cases was significantly higher than in T1 cases (median 241 cells/mm^2^ and 97 cells/mm^2^, respectively, *p* < 0.001) (Table 2, Fig. 3a). In the whole analyzed specimen area (epithelial + stroma) of tumor resection samples the Gal3 expression in T2 cases was also significantly higher than in T1 cases (median 302 cells/mm^2^ and 116 cells/mm^2^, respectively, *p* = 0.002) (Table 2, Fig. 3b).

**Table 2:** Galectin 3 (Gal3) cell count (cells/mm2) and Gal3/CD68 expression ratio in oscc tumor resection specimens

Gal3 expression in tumor resection specimens									
Marker		Gal3 epithelial		Gal3 epithelial + stroma		Ratio Gal3/CD68 epithelial		Ratio Gal3/CD68 epithelial + stroma	
		Median	SD	Median	SD	Median	SD	Median	SD
T-Status	n								
T1	14	97	48	116	129	0.30	0.29	0.24	0.27
T2	20	241	127	302	169	0.73	0.79	0.58	0.64
*p*-value		0.000		0.002		0.002		0.010	
N-Status	n								
N0	19	126	89	169	164	0.71	0.71	0.42	0.50
N+	15	234	156	287	182	0.59	0.78	0.57	0.66
*p*-value		0.030		0.106		0.943		0.337	
L-Status	n								
L0	25	180	138	195	190	0.71	0.74	0.45	0.65
L1	9	203	115	223	143	0.59	0.74	0.46	0.26
*p*-value		0.892		0.999		0.834		0.595	
Pn-Status	n								
Pn0	21	130	134	191	206	0.48	0.68	0.42	0.67
Pn1	10	199	138	232	134	0.66	0.86	0.56	0.44
*p*-value		0.452		0.959		0.942		0.679	
Grading	n								
G2	26	155	132	194	169	0.52	0.46	0.45	0.44
G3	6	254	142	232	246	1.31	1.11	0.56	0.96
*p*-value		0.422		0.528		0.008		0.183	

Table shows the Galectin 3 (Gal3) cell count (positive cells/mm^2^) and the ratio of Gal3 positive cells and CD68 positive cells in oscc tumor resection specimens depending on histomorphologic parameters (T-, N-, L-, Pn-status, grading). Results for the epithelial tumor compartment and the whole analyzed area (epithelial + stroma) are given. Values represent the median, standard deviation (SD) and p-value (ANOVA)

The Gal3/CD68 expression ratio in the epithelial fraction of T2 oscc tumor resection specimens was significantly higher than in T1 cases (median value 0.73 and 0.30, respectively, *p* = 0.002) (Table 2, Fig. 3c). The whole analyzed specimen area (epithelial + stroma) in T2 oscc tumor resection specimens also showed a significantly higher Gal3/CD68-ratio than in T1 cases (median value 0.58 and 0.24, respectively, *p* = 0.010) (Table 2, Fig. 3d).

The epithelial compartment of oscc tumor resection specimens in cases with lymph node metastases (N+) revealed a significantly higher Gal3 cell count than N0 cases (median 234 cells/mm^2^ and 126 cells/mm^2^, respectively, *p* = 0.030) (Table 2, Fig. 3e).

The Gal3/CD68-ratio in the epithelial compartment of G3 oscc tumor resection specimens was significantly higher compared to G2 cases (median value 1.31 and 0.52, respectively, *p* = 0.008) (Table 2, Fig. 3f).

There was no significant association apparent between Gal3 expression and L-status or Pn-status in oscc tumor resection specimens.

## Discussion

### Gal3 expression in oscc biopsy and tumor resection specimens

The current study revealed an association between Galectin 3 (Gal3) expression in oral squamous cell carcinoma (oscc) tissue and histomorphologic parameters of tumor progression (T-, N-, L-, Pn-stage, grading). In biopsy and tumor resection specimens, the number of Gal3 positive cells as well as the ratio between Gal3 expressing cells and CD68 positive macrophages was higher in T2 oscc cases compared to T1 cases (Figs. 2 and 3). Considering the tumor promoting and immunosuppressive function of Gal3 [23, 25], this finding could implicate that local immunosuppression promotes increasing tumor growth and consequently size. The correlation between tumor size (T-status) and Gal3 expression resp. Gal3/CD68 ratio was seen in the epithelial tumor compartment as well as the whole analyzed specimen area including stroma and epithelium (Figs. 2a-d and 3a-d). In a previous study in the patient cohort analyzed here, no association was detected between T-status and the density of CD68 positive macrophages [3].

An association between Gal3 expression and T-status was seen in biopsies and in tumor resection specimens. As previously reported, there are immunologic changes in tumor tissue occurring during the time interval between the diagnostic biopsy and the definitive tumor resection [24]. The association between T-status and Gal3 expression in biopsies and tumor resection samples observed in the current report (Fig. 2a-d and 3a-d) indicates that Gal3 might be a possible robust biologic parameter that is not relevantly influenced by biopsy-derived tissue trauma.

Additionally, the current analysis showed an association of higher Gal3 expression in tumor resection specimens with the occurrence of lymph node metastases (N+) (Fig. 3e). A high Gal3/CD68 ratio correlated with higher grading (G3) in oscc tumor resection specimens (Fig. 3f). In biopsies, the Gal3 expression and the Gal3/CD68 ratio was associated with the presence of lymph vessel infiltration (L1) (Fig. 2e-f). As the N-status, the tumor grading and the L-status are important parameters of malignant behavior, the association of Gal3 expression with these parameters underlines a possible tumor-promoting role of Gal3 in oscc.

### Gal3 mediated immunosuppression

Gal3 can interact with macrophages and modulate macrophage polarization [7, 26]. M2 polarized macrophages show a significantly increased Gal3 expression and secretion [7, 26, 27]. In contrast, induction of M1 polarization in macrophages leads to an inhibition of Gal3 expression compared to unstimulated monocytes [7].

Additionally, Gal3 itself can shift macrophage polarization towards M2 polarized cells. In murine cells, Gal3 deficiency prevented the M2 polarization of macrophages while there was no influence observed on the induction of M1 polarization [7]. Furthermore, M2 polarization can be prevented by the use of small inhibitory RNA (siRNA) for Gal3 or for the Gal3 receptor CD98. CD98 shows strong expression on macrophages and leads to the activation of the second messenger phosphatidylinositol 3-kinase (PI3K) [7]. PI3K activation is a relevant pathway for the induction of M2 macrophage polarization. Hence, Gal3 can induce PI3K activation via CD98 and thereby induce M2 polarization of macrophages [7]. Consequently, there might be a Gal3 triggered positive feedback loop for M2 polarization of macrophages as Gal3 contributes to the induction of M2 polarization and M2 macrophages show an increased Gal3 production [7].

This loop could be targeted by pharmacologic inhibition of Gal3 e.g. with citrus pectin [25] or by blocking CD98 [7]. As we detected an association of Gal3 with parameters of malignancy in oscc, targeting the Gal3 – CD98 – PI3K pathway could be a potential immune modulating treatment option for oral cancer patients.

These findings suggest the possibility of an increased Gal3 production in the context of a malign disease leading to an increased degree of M2 polarization of macrophages. On the other hand, it would be conceivable that macrophage-derived Gal3 itself leads to a promotion of malign transformation and metastatic spread of oscc tumor cells. Besides its influence on macrophage polarization, Gal3 can inhibit T-cell activation by destabilizing the immunologic synapse [28]. Additionally, Gal3 can inhibit CD8 positive cytotoxic T-cells in an LAG-3 dependent manner and thereby contribute to local immune tolerance and tumor promotion [29]. Consequently, Gal3 could contribute to an immunosuppressive local tumor environment at the level of macrophages and T-cells which could be targeted by Gal3 inhibitors like citrus pectin [25].

### Lineage of Gal3 positive cells

The current study reveals an association between tumor size (T-status) and parameters of malignancy (L-status, grading) with the Gal3/CD68 ratio. In most cases the detected Gal3/CD68 ratio was smaller than 1, meaning that less Gal3-positive cells than CD68-positive macrophages were present in the specimens (Tables 1 and 2). An association between the absolute cell count of CD68-positive macrophages in the epithelial tumor compartment and the occurrence of lymph node metastases was already published in a previous report of our group [3].

Cells with high Gal3 expression were detected in the epithelial tumor compartment and in the tumor stroma (Fig. 1). This indicates that a relevant proportion of the highly Gal3 expressing cells in oscc specimens are no tumor cells but stroma cells or tumor infiltrating immune cells.

One part of the detected Gal3 expressing cells might be M2 polarized macrophages. An association between M2 polarization of macrophages and high Gal3 expression is already shown [7, 26, 27]. A previous report analyzing cervical squamous cell carcinomas revealed that many intratumoral Gal3 expressing cells are CD163 positive M2 polarized macrophages [27]. However, the Gal 3 positive cell population identified in the current study might also include cancer stem cells [30, 31].

## Conclusion

High Gal3 expression as well as a high Gal3/CD68 ratio correlated with tumor size and parameters of malignancy. Our data indicate that Gal3 has a negative tumorbiological influence on oral squamous cell carcinomas (oscc) and we hypothesize that it might exert this influence via a modulation of macrophage polarization. Further studies are needed to identify the exact lineage of the Gal3 expressing cells in oscc tumor epithelium and stroma and the mechanisms by which Gal3 influences macrophage polarization and immune surveillance. Gal3 might serve as a target of immune therapy in oscc as Gal3 inhibitors like citrus pectin are available.

## Abbreviations

Gal3: Galectin 3
LAG-3: Lymphocyte-activation gene 3
M1: M1 polarized macrophages
M2: M2 polarized macrophages
oscc: Oral squamous cell carcinoma
PI3K: Phosphatidylinositol 3-kinase
SD: Standard deviation
siRNA: Small inhibitory RNA
